# Feature Extraction and Classification of Citrus Juice by Using an Enhanced L-KSVD on Data Obtained from Electronic Nose

**DOI:** 10.3390/s19040916

**Published:** 2019-02-21

**Authors:** Wen Cao, Chunmei Liu, Pengfei Jia

**Affiliations:** 1 School of Information Engineering, Southwest University of Science and Technology, Mianyang 621010, China; caowen@swust.edu.cn (W.C.); liuchunmei@swust.edu.cn (C.L.); 2 College of Electronic and Information Engineering, Southwest University, Chongqing 400715, China

**Keywords:** electronic nose, citrus juice aroma, sparse coding, L-KSVD, kernel function

## Abstract

Aroma plays a significant role in the quality of citrus fruits and processed products. The detection and analysis of citrus volatiles can be measured by an electronic nose (E-nose); in this paper, an E-nose is employed to classify the juice which is stored for different days. Feature extraction and classification are two important requirements for an E-nose. During the training process, a classifier can optimize its own parameters to achieve a better classification accuracy but cannot decide its input data which is treated by feature extraction methods, so the classification result is not always ideal. Label consistent KSVD (L-KSVD) is a novel technique which can extract the feature and classify the data at the same time, and such an operation can improve the classification accuracy. We propose an enhanced L-KSVD called E-LCKSVD for E-nose in this paper. During E-LCKSVD, we introduce a kernel function to the traditional L-KSVD and present a new initialization technique of its dictionary; finally, the weighted coefficients of different parts of its object function is studied, and enhanced quantum-behaved particle swarm optimization (EQPSO) is employed to optimize these coefficients. During the experimental section, we firstly find the classification accuracy of KSVD, and L-KSVD is improved with the help of the kernel function; this can prove that their ability of dealing nonlinear data is improved. Then, we compare the results of different dictionary initialization techniques and prove our proposed method is better. Finally, we find the optimal value of the weighted coefficients of the object function of E-LCKSVD that can make E-nose reach a better performance.

## 1. Introduction

Citrus juice is famous for its rich nutrition, delicious taste and aroma-make, and aroma is a significant factor that affects the quality of citrus fruits and processed products. It is important for us to study the aroma of citrus processed products due to the fact that characteristics of aroma can be a standard for testing in the field of food. When tasting a citrus, we can use multiple senses, such as aroma and taste, to feel the stimulus. Also, flavor is a crucial factor in determining what citrus quality and nutrition value is. At present, the main method of testing the citrus quality is sensory analysis and precision instrumental.

Sensory analysis is used in traditional processing extensively. Although this test method is extremely consumer-friendly, people are too emotional to have a similar standard of the same juice. At the same time, various degrees of fatigue problems will affect experimenters’ sensory analysis, which may cause the experimenters to come to inaccurate or even wrong conclusions and make the results subjective and unscientific.

Methods for precision instrumental analysis include gas chromatography (GC), gas chromatography-olfactometry (GC-O), gas chromatography-mass spectrometry (GC-MS), etc. [1,2]. The analysis of precision instruments has become the main analytical test method in the scientific investigation because of its high repeatability and the results of analysis being objective and scientific. However, there are still some drawbacks using an analysis of precision instruments method. First, it cannot be applied well to the production and living, which is restrictive to a full research platform with low cost. Secondly, the preprocess steps of a precision instrument analysis are complicated, and the instrument is inconvenient to operate, which means it is difficult for operators to meet strict skill requirements. Last but not least, consumers pay more attention to the overall feeling rather than to the specific ingredients of the juice when they smell the juice.

The evaluation of the odor for juice is important, so an efficient and low-threshold technique of the actual production is in demand, which can focus especially on the overall feeling of the product and analyze the attributes of the product scientifically and objectively.

An electronic nose (E-nose) is a device which is designed like the biological olfactory device to analyze the gas/odor [3,4]. It combines an array of gas sensors and series-related intelligent algorithms. Through the gas sensor array, an E-nose can gain response curves of the sensors to the target gas/odor, and then, intelligent algorithms are used to deal with the response to identify the gas/odor, which includes a single or complex component(s) [5]. Comparing the sensory analysis with the precision instrumental analysis, an E-nose is considerable, low cost and fast detection. It has been applied to various fields such as wound detection [6], disease detection [7], food engineering [8,9,10], environmental control [11], explosive detection [12], agriculture [13,14] and so on. 

Reference [15] validated an E-nose to identify “La France” pears of different maturity and compared them by using chemical analysis methods (GC and GC-MS). The result shows that the judgment result of an E-nose is very consistent. An E-nose was used to study the quality of citrus and apple by Corrado Di Natale et al., and they proved that an E-nose has enough sensitivity and resolution to distinguish among the various classes and to correctly predict the number of defects (for apples) and storage days (for oranges). Moreover, References [16,17,18] proved that E-nose can be used to identify the maturity and quality of various fruits.

Feature extraction is the first step of the sensor signal processing and affects the accuracy of the pattern recognition a lot [19]. To get the utmost out of the experiment data, classifiers are used for learning a classification function or constructing a classification model based on the original data. Feature extraction and pattern recognition play important roles in helping an E-nose get a high recognition rate, and they are two independent steps traditionally. 

Extracting more features from an E-nose response can help improve the classification accuracy. In the traditional way, we would like to get the maximum value of the steady-state response of sensors to construct the original feature matrix and use this matrix to classify, but its accuracy is not ideal. To get a better performance, some classical algorithms are used for reprocessing the original feature matrix. Principal component analysis (PCA) is a method to find several comprehensive indicators to represent many original features, so that these comprehensive indicators reflect the original variables as much as possible, and they are not related to each other [20]. Independent component analysis (ICA) [21] is a statistical method used to convert observed multidimensional vectors into statistically independent components for eliminating the redundancy of the original data. These algorithms help the classifier improve accuracy. However, PCA and ICA are both linear methods of which the performance is not very ideal when used to deal with the nonlinear data. The kernel PCA (KPCA) is a nonlinear method which finds a computationally tractable solution through a simple kernel function that intrinsically constructs a nonlinear mapping from the input space to the high-dimensional space and then performs a nonlinear PCA in the high-dimensional space [22].

When the feature matrix is obtained, it will be put into the classifier. The most common classifiers used in an E-nose is the linear discriminant analysis (LDA) [23], radial basis function neural network (RBFNN) [24] and support vector machine (SVM) [25]. LDA is a linear classify, and its target during the training process is to shorten the distance between the samples from the same class and largen the distance of samples from the different classes. With the help of the kernel function, LDA can classify the nonlinear data. RBFNN using a radial basis function as its nonlinear mapping function is not easy to fall into the local optimum, but the time it spends during the training process is a little long. SVM is a widely used classifier in an E-nose. Its training target is not only the highest classification accuracy of the training data set but also the highest classification accuracy of the test data set, and it has a good generalization ability.

We cannot help but think that if we unify the process of feature extraction and the classifier, as a joint indicator, can we get a better recognition rate. Inspired by this, we find the label consistent KSVD (L-KSVD), which combines the feature extraction with the classification. KSVD is a dictionary learning algorithm for creating a dictionary for spare representations via a singular value decomposition approach. KSVD is a generalization of the k-means clustering method, and it works by iteratively alternating between sparse coding the input data based on the current dictionary and updating the atoms in the dictionary to better fit the data [26]. The innovation of this paper is pointed out as follows:

(a) The traditional L-KSVD cannot handle the problem of nonlinear data very well, and the kernel function is adopted in this paper to help L-KSVD deal with the nonlinear data obtained by the E-nose.
(b) Choosing a proper dictionary is the first and most important step of L-KSVD, and a novel dictionary initialization method is proposed according to the data characteristics of the E-nose. With the help of the Enhance Quantum-behaved Particle Swarm Optimization (EQPSO), this method generates random numbers in binary and uses the recognition rate as a fitness function to decide which sensor response will be used to initialize the dictionary.
(c) The weighted coefficients of the objective function of L-KSVD have a bigger impact on the classification accuracy, so these coefficients are standardized and then optimized with the help of EQPSO in this paper.

In the rest of this paper, we present the experiment of this paper in Section 2 and introduce the proposed L-KSVD in Section 3. The results and discussion will be presented in Section 4. Finally, the conclusions are drawn in Section 5.

## 2. Materials and Methods

**Figure 1:**
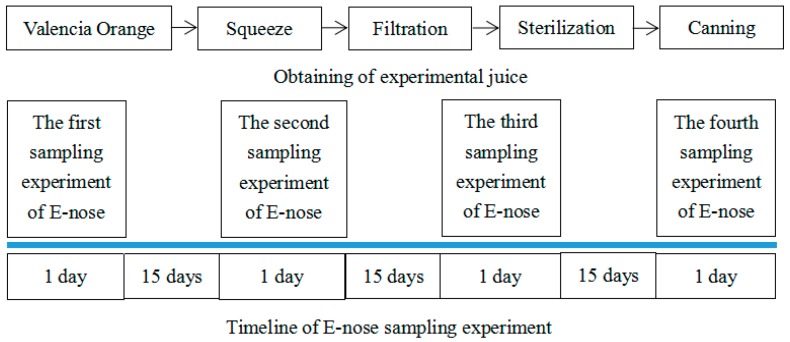
The obtainment of experimental juice and the timeline of an E-nose sampling experiment.

In this project, we use the cold-pressed technology to process the Valencia oranges with the same maturity to obtain orange juice: 50 kg oranges are sued in this project, and the shape and size of each orange is almost same. These oranges come from 10 trees of similar age and growth in the same orchard and are picked at the same position of each tree (about 5 kg oranges from one tree). During the process of obtaining the experimental juice, filtration, sterilization and canning are implemented in turn. Then, the juice is stored in a storage tank (its volume is 30 L), and nitrogen is filled in the top of the storage tank to be isolated from air, and the air pressure of the tank is 202.6 KPa. On the bottom of the tank, there is a tap which is used for the sampling of the orange juice. Inside the tank, there is a blender, and every time before the sampling, we turn it on for 1 min to stir the orange juice thoroughly in case the orange juice might delaminate or precipitate, and the blender spins on its axis once every 1 s. The sampling is proceeded every 15 days, and more information of the above description can be found in Figure 1. After each sampling, the orange juice is put into 4 sterilized and identical glass vessels, and each of them contains 500 mL of orange juice. 

**Figure 2:**
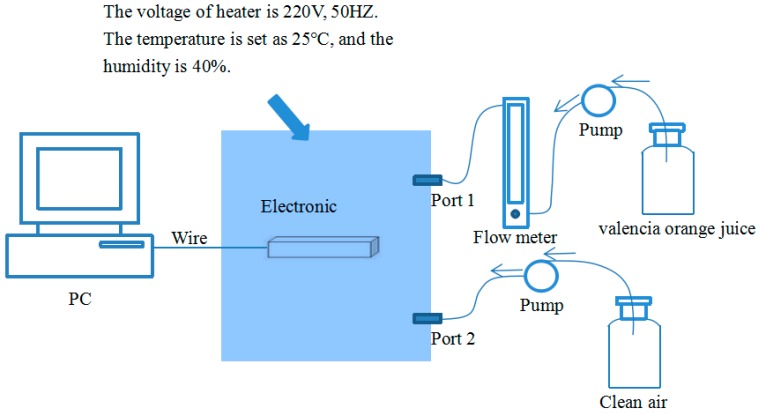
A schematic diagram of the experimental system.

The analysis of the orange juice aroma components is shown in Table 1, and the schematic diagram of the experimental E-nose system is shown in Figure 2. 

**Figure 3:**
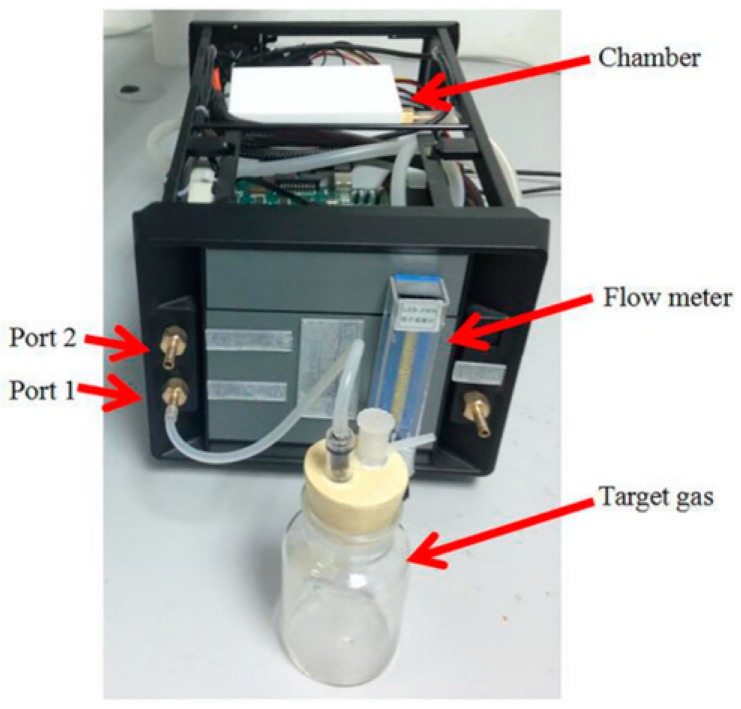
A practical E-nose system.

During each sampling experiment of the E-nose, we set the temperature and humidity of the chamber of the E-nose at 25 °C and 40%, respectively. The rules used when we design and construct the sensor array are (a) the sensor array can respond to all classes of the juice odor; (b) each sensor has its own interesting odor and can also respond to other odors of juice; and (c) a high-performance cost ratio and an easy purchase are requirements. Table 2 lists the sensors selected by us and their sensitive characteristics. The gas sensor array was located in the Teflon chamber with a volume of 0.24 L, and the flow rate can be controlled by a flowmeter and a micro pump with a set value of 0.08 L/min. The practical E-nose system designed by us is shown in Figure 3.

In the rubber stopper, there are two holes with two thin Teflon tubes inserted: one Teflon tube fixed as close as possible to Valencia orange juice. The output gas from the tube containing VOCs of the Valencia orange juice flows out of the bottle and then flows into the chamber through a Teflon tube. As for each sampling experiment, the following three stages should be performed:

Step (a) expose all sensors to clean air for 5 min to obtain the baseline.
Step (b) introduce the target gas into the chamber for 7 min.
Step (c) exposed the sensor array to clean air for 5 min again to clean the sensors and restore the baseline.

In each experiment, each cup of orange juice is tested by an E-nose 6 times so that 24 sample data are recorded in total. After 4 sampling experiments, the data set of this project, which contains 96 sample data, is obtained.

**Figure 4:**
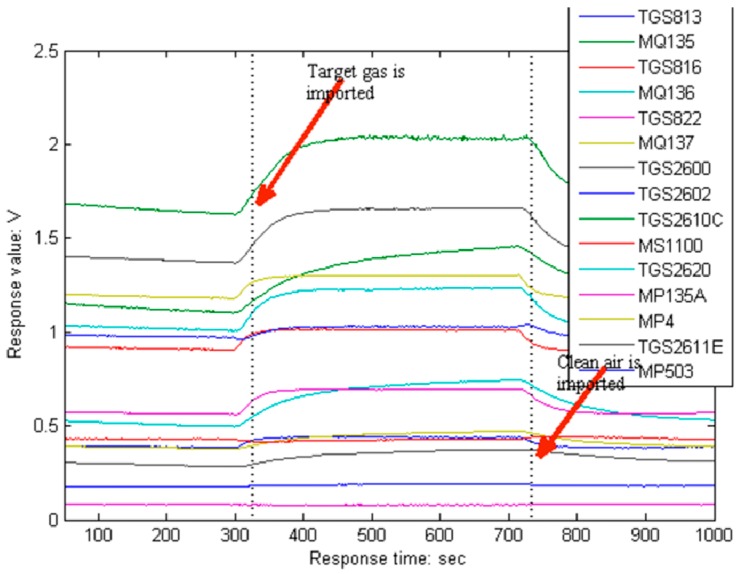
The response of the sensor array. Note: Strictly speaking, the response of the metal oxide sensor (MOS) should be the resistance value, and the response of the vertical coordinate in this diagram is the voltage value, which is the voltage value that we use in the circuit (designed by ourselves) to transform the change of resistance value to the change of voltage value. The resistance of the sensor is different when the gas environment is different, and the voltage value is also different, so we take the voltage value as the response value of sensors in this paper.

The sensors used by the E-nose of this paper are all metal oxide sensor (MOS). With a MOS, when its resistance wire contacts different gases, its resistivity will change, which will lead to the change of its resistance value. The E-nose hardware circuit designed in this paper can transform the change of resistance value to the change of voltage value, and then, we record this voltage value. Therefore, the voltage value is taken as the response value of the sensor in this paper. For this voltage value, we first use analog circuits to filter and amplify it, and then, the response of the sensor array is sampled by a 14-bit data acquisition system (DAS) which is bought from the market, and the output signal of DAS is sent to the computer by a USB data line. Figure 4 illustrates the response of the sensors when Valencia orange juice odor is introduced into the chamber. We can see that each response curve rises obviously from the fifth minute when the target gas begins to pass over the sensor array and recovers to the baseline after the twelfth minute when clean air is conveyed to wash the sensors. 

Then the maximum value of the steady-state response of sensors is extracted to create the feature matrix of the E-nose. There are 96 samples in this matrix, and the dimension of each sample is 15. We randomly select 2/3 samples of each gas to establish the training data set, and the rest are used as the test data set. This feature matrix is called the original feature matrix, and it is the input of the proposed KSVD algorithm. 

## 3. Methodology

### 3.1. KSVD and L-KSVD

KSVD is a generalization of the k-means clustering method, and it works by iteratively alternating between sparse coding the input data based on the current dictionary and updating the atoms in the dictionary to better fit the data. KSVD can be found widely used in applications such as image processing, audio processing, biology and document analysis. KSVD learns a shared dictionary, which optimizes the following objective function:

(1) $$\arg\underset{D,Z}{\min}{\left\|Y-DX\right\|}_{F}^{2}\;s.t.\;\forall i,\;{\left\|x_{i}\right\|}_{0}\leq T$$

where $Y=\left[y_{1},y_{2},\cdots ,y_{N}\right]\in R^{n\times N}$ are *N* input signals and each is in the n dimension; $D=\left[d_{1},d_{2}\cdots ,d_{K}\right]\in R^{n\times K}$ (K >> n, making D over-complete) is a dictionary with atoms; $X=\left[x_{1},x_{2},\cdots ,x_{N}\right]\in R^{K\times N}$ are *N* sparse codes of input signals Y; and T is a constant, which controls the number of nonzero elements in $x_{i}$ less than T.

Solving the minimization of Equation (1) by a two-step iterative algorithm: Firstly, the dictionary is fixed, and the sparse coefficients X can be found. This is the problem of sparse coding, which can be solved by an orthogonal matching pursuit (OMP) [27,28]. Secondly, while fixing all other atoms in D, the sparse coefficient matrix X is fixed and dictionary *D* is updated one atom at the same time.

For each atom, $d_{k}$ and the corresponding $k_{th}$ row of coefficient matrix X denoted by $x_{T}^{k}$ define the group of samples that use this atom as $\omega_{k}=\left\{i|1\leq i\leq N,x_{T}^{k}\left(i\right)\neq 0\right\}$. The error matrix computes $E_{k}=Y-\sum_{i\neq k}d_{i}\times x_{T}^{i}$, restricts $E_{k}$ by choosing only the columns in $\omega_{k}$ and obtains $E_{k}^{R}$. Then, the following problem is solved:

(2) $$\arg\underset{d_{k},z_{T}^{k}}{\min}{\left\|E_{k}^{R}-d_{k}\times x_{T}^{k}\right\|}_{F}^{2}$$

where a singular value decomposition (SVD) is performed and $E_{k}^{R}=U\Delta V^{T}$.

A label consistent KSVD (L-KSVD) algorithm to learn a discriminative dictionary for sparse coding was presented by Zhuojin Jiang et al. This algorithm learns a single over-complete dictionary and an optimal linear classifier jointly. It yields dictionaries so that feature points with the same class labels will have similar sparse codes. 

In order to use class labels of training data, the associating label information with each dictionary item (columns of the dictionary matrix) enforces discriminability in sparse codes during the dictionary learning process [29]. 

The aim is to leverage the supervised information of input signals to learn a reconstructive, discriminative dictionary and to include the classification error as a term in the objective function for dictionary learning; each dictionary item is chosen to represent a subset of the training signals ideally from a single class: in that case, each dictionary item $d_{k}$ can be associated with a particular label. Thus, there is an explicit correspondence between the dictionary items and the labels. Then, L-KSVD focuses on the effects of adding a label-consistent regularization term, subsequently, to learn both more balanced reconstructive and discriminative power, making the objective function with a joint classification error and label-consistent regularization term. 

The performance of the linear classifier must be based on the discriminability of x (the input sparse codes). To accept the discriminative sparse codes that have the learned D, an objective function of dictionary construction is defined as follows:

(3) $$<D,W,A,X>=\arg\underset{D,W,A,X}{\min}{\left\|Y-DX\right\|}_{2}^{2}+\alpha {\left\|Q-AX\right\|}_{2}^{2}+\beta {\left\|H-WX\right\|}_{2}^{2}\;s.t.\forall i,{\left\|x_{i}\right\|}_{0}\leq T$$

where α dominates the reconstruction and label-consistent regularization, ${\left\|H-WX\right\|}_{2}^{2}$ represents the classification error, and W is the classifier parameters to make the classification dictionary optimal. L-KSVD makes the linear predictive classifier f(x;W)=Wx, with $Q=\left[q_{i}^{1}\ldots q_{i}^{K}\right]\in R^{K\times N}$ as the input signal Y of the discriminative sparse codes of the classification, denote the discriminative sparse code $q_{i}={\left[q_{i}^{1}\cdots q_{i}^{K}\right]}^{t}={\left[0\ldots 1,1,\ldots 0\right]}^{t}\in R^{K}$ consistent with $y_{i}$ (input signal), when $q_{i}$ are nonzero values on these indices where $y_{i}$ and $d_{k}$ (dictionary item) use a communion label. For instance, It can be assumed $D=\left[d_{1}\ldots d_{6}\right]$ and $Y=\left[y_{1}\ldots y_{6}\right]$, where $y_{1}$, $y_{2}$, $d_{1}$ and $d_{2}$ are from class 1; $y_{3}$, $y_{4}$, $d_{3}$ and $d_{4}$ are from class 2; and $y_{5}$, $y_{6}$, $d_{5}$ and $d_{6}$ are from class 3. Q could be defined as

(4) $$Q\equiv \left[\begin{array}{l} 110000\\ 110000\\ 001100\\ 000011\\ 000011 \end{array}\right]$$

*A* is a linear transformation matrix, and L-KSVD identifies g=(x;A)=Ax; in the sparse feature space $R^{K}$, the original sparse code x is transformed to the most discriminative one. ${\left\|Q-AX\right\|}_{2}^{2}$, on behalf of the discriminative sparse-code error, performs *X* approximate discriminant sparse codes. It forces the signals from the same class to have very similar sparse representations and uses simple linear classifiers to achieve a good classification performance. $H=\left[h_{1\cdots }h_{N}\right]\in R^{m\times N}$ is a class label of *Y*. $h_{i}={\left[0,0\ldots 1\ldots 0,0\right]}^{t}\in R^{m}$ is a label vector the goes to the input signal $y_{i}$, and the nonzero position indicates the location of the classes. α and β are scalars for controlling the relative contribution of the corresponding items.

Dictionaries learned with the method will adapt to the structure of the training data (resulting in a good representation of the strict sparse constraints on each member of the set) and will make discriminatory sparse codes *X* disregard the dictionary size. These sparse codes can be directly used by classifiers, such as in Reference [30]. The discriminating characteristics of sparse codes x are very important for the performance of linear classifiers.

The brief introduction of KSVD and L-KSVD are shown above. When we apply L-KSVD to solve the problem of data processing of an E-nose, we find that several problems need to be solved: 

The distribution of data gained by E-nose data is nonlinear, so the effect of KSVD/L-KSVD is not ideal when they are used to process the data directly; since the sensor of an E-nose is cross-responsive, the data is redundant. When the dictionary of KSVD/L-KSVD is initialized, different sensor responses will be selected and the corresponding recognition rate will be different. The weighted coefficients of three parts of the L-KSVD objective function will determine the influence of each part on the final result.

During Section 3.2, Section 3.3 and Section 3.4, we will solve the above three problems, respectively. The L-KSVD enhanced by techniques from Section 3.2, Section 3.3 and Section 3.4 is called E-LCKSVD.

### 3.2. Kernel Function

L-KSVD for sparse coding has contributed a lot, which lies in explicitly integrating the discriminative sparse codes and a single predictive linear classifier into the objective function for dictionary learning. However, this algorithm cannot handle the problems of the nonlinear data very well. As nonlinear dynamical systems which are difficult to solve in mathematics and science, based on pattern recognition theory, the low-dimensional space linear inseparable model through nonlinear mapping to a high-dimensional feature space may be linearly separable, but if we use this technique in a high-dimensional space classification or regression directly, there is a big obstacle named dimension disaster which will exist in high-dimensional feature space operations. 

It has been proved that the kernel function can be used to solve this problem effectively. A kernel is a nonnegative real-valued integrable function; it is desirable to define the function for most applications to satisfy two additional requirements: normalization and symmetry. As we know, several types of kernel functions are commonly used in many fields, especially in a support vector machine (SVM). SVM maps the sample space to a feature space of high or even infinite dimensions through nonlinear mapping so that the nonlinear separable problems in the original sample space are transformed into linearly separable problems in the feature space. As for the problems of classification and regression, it is likely that the sample set cannot be processed linearly in the lowdimensional sample space, but the linear partition (or regression) can be achieved through a linear hyper plane in the high-dimensional feature space. As the linear learning machine is established in the high-dimensional feature space, it does not increase the complexity of the calculations and avoids the dimensional disaster to some extent compared with the linear model. All of this is due to the kernel function expansion and computational theory. 

The kernel function can be combined with different algorithms to form a variety of different methods based on kernel function technology, and the design of these two parts can be carried out separately. The combination of L-KSVD and the kernel function can solve the nonlinear problem of the E-nose.

In this paper, firstly, we use the RBF kernel to map the data of an E-nose and then run KSVD/L-KSVD, namely, in the high-dimensional space. The expression of RBF kernel is

(5) $$k(x_{i},x_{j})=\exp(-\frac{{\left\|x_{i}-x_{j}\right\|}^{2}}{\sigma^{2}})$$

where σ is the scale factor which determines the distribution of the data mapped to the high-dimensional space, so it is a very important parameter. In this paper, an optimization algorithm named EQPSO is used to set its value.

### 3.3. Dictionary

Choosing a proper dictionary is the first and most important step of the sparse representation based on classifications with encouraging results [31]. Especially, dictionaries learned from training data obtain researchers’ attentions because the learned dictionaries usually lead to a better representation and achieve much success in classification.

The goal of dictionary learning is to learn an over-complete dictionary matrix $D\in R^{n\times K}$ in which K contains signal-atoms (in this notation, columns of D). A signal vector $y\in R^{n}$ can be represented, sparsely, as a linear combination of these atoms; to represent y, the representation vector x should satisfy the exact condition y=Dx, or the approximate condition y≈Dx, made precise by requiring that ${\left\|y-Dx\right\|}_{p}\leq \varepsilon$ for some small value ε and some $L_{p}$ norm. The vector $x\in R^{K}$ contains the representation coefficients of the signal y. Typically, the norm p is selected as $L_{1}$, $L_{2}$ or $L_{\infty }$.

If n<K and *D* is a full-rank matrix, there are an infinite number of solutions for the representation problem. Then, constraints should be set on the solution. Also, to ensure sparsity, the solution with the fewest number of nonzero coefficients is preferred, which means the sparsity representation is the solution of either $(P_{0})\underset{x}{\min}{\left\|x\right\|}_{0}$ subject to y=Dx or $(P_{0,\varepsilon })\underset{x}{\min}{\left\|x\right\|}_{0}$ subject to ${\left\|y-Dx\right\|}_{2}\leq \varepsilon$, where the ${\left\|x\right\|}_{0}$ counts the nonzero entries in the vector x.

Minimizing the reconstruction error and satisfying the sparsity constraints to achieve the construction of D: Although the random dictionary initialization has an outstanding effect on images of compressing and restorations, it is not good enough to identify gas in an E-nose due to the fact that the cross-responsiveness of the sensor array, that is, each sensor, will respond to the same gas, which means that the response of the sensor is overlapping and redundant. 

To select the best combination of sensors, we use random binary number to filter proper dictionary initialized atoms: 1 represents this sensor is selected, and 0 represents not. Remove the information which is redundant, and filter the characteristic representative data in this way. The binary parameter is provided by EQPSO (shown in Section 3.5), and the way to generate the random binary number can be found in Section 3.5.

### 3.4. Weighted Coefficients

At first, we use a linear predictive classifier f(x;W) = Wx. An objective function for learning a dictionary D having both reconstructive and discriminative power can be defined as follows:

(6) $$\begin{array}{cc} <D,W,A,X> & =\;\arg\;\underset{D,W,A,X}{\min}{\left\|Y-DX\right\|}_{2}^{2}+\alpha {\left\|Q-AX\right\|}_{2}^{2}\\ & \;+\beta {\left\|H-WX\right\|}_{2}^{2}\;s.t.\forall i,\;{\left\|x_{i}\right\|}_{o}\leq T, \end{array}$$

In this paper, Equation (6) is changed by us to the following:

(7) $$\begin{array}{cc} <D,W,A,X> & =\;\arg\;\underset{D,W,A,X}{\min}\alpha {\left\|Y-DX\right\|}_{2}^{2}+\beta {\left\|Q-AX\right\|}_{2}^{2}\\ & \;+\gamma {\left\|H-WX\right\|}_{2}^{2}\;s.t.\forall i,\;{\left\|x_{i}\right\|}_{o}\leq T \end{array}$$

where α + β + γ = 1 and α, β, γ ε (0,1). We define this process as normalization processing. In Equation (7), the value of α, β and γ changes the proportion of each part in the whole expression; it is a little bit more intuitive to see which part is going to have a bigger impact on the outcome result of the recognition rate. We can see the classification accuracy of E-LCKSVD is very different when the values of α and β are different from Table 3, so the values of α, β and γ have great influences on the classification accuracy. Some optimization technique must be employed to set their value.

### 3.5. EQPSO and the Optimization Problem of the Proposed E-LCKSVD

#### 3.5.1. PSO, QPSO and EQPSO

Particle swarm optimization (PSO) is an evolutionary computation algorithm based on the swarm intelligence theory. This swarm intelligence algorithm for continuous searching space problems is widely used for its simple programming and fast convergence speed and is used in Reference [32]. Among them,

(8) $$P_{id}(t)=\frac{c_{1}r_{1d}(t)P_{id}(t)+c_{2}r_{2d}(t)P_{gd}(t)}{c_{1}r_{1d}(t)+c_{2}r_{2d}(t)},\;1\leq d\leq D,$$

and

(9) $$\phi_{d}(t)=\frac{c_{1}r_{1d}}{c_{1}r_{1d}(t)+c_{2}r_{2d}(t)},$$

where *t* is the current iteration number of the algorithm, $r_{1d}(t)$ and $r_{2d}(t)$ are random numbers in [0,1]; *P_i_* is the current optimal position of the particle; and *P_g_* is the global optimal position of the group. However, in a classic PSO, the particle search process is realized in the form of orbit and the particle’s flight speed is limited. Therefore, in the search process, the particle search space is limited to a limited search space, which cannot cover the whole feasible search space. A general PSO cannot guarantee convergence to the global optimal solution with probability 1, which is the deficiency of PSO.

In order to solve this shortcoming of PSO, quantum-behaved PSO (QPSO) has been proposed [33]. QPSO combine PSO with quantum mechanics. QPSO has great advantages in terms of search ability, convergence speed, accuracy and solving robustness. Compared with the other algorithm, one of the biggest characteristics of QPSO is the simple calculation and few control parameters. It is superior to the general PSO not only in search ability but also in its accuracy, and QPSO can guarantee the global convergence with probability 1. 

The wave function ϕ(X,t) is used to determine the state of each particle and the definition of the average optimal position which is the center of the optimal position of all particles. We can get an updated Equation (10) for the position of the particle:

(10) $$\begin{array}{l} x_{ij}(t+1)=p_{ij}(t)\pm \alpha \cdot \left|M_{ij}(t)-x_{ij}(t)\right|\cdot \ln[\frac{1}{u_{ij}(t)}]\\ u_{ij}(t)\sim U(0,1) \end{array}$$

where α is called the compression expansion factor to regulate the rate of convergence of particles. However, when the number of iterations is not infinite, QPSO cannot guarantee to find the global optimal value. In practice, the number of iterations is always limited.

Enhanced QPSO (EQPSO) [34,35] is an improved QPSO algorithm, which can ensure to find the value closest to the optimal value in the case of limited iteration times. It has a better ability of making particles multifarious at the early iteration’s stage and performing in local searching ability at the later stage of iteration. Meanwhile, the convergence speed of EQPSO is faster than other considered PSOs, such as PSO and QPSO. Therefore, EQPSO is selected as the optimization algorithm in this paper.

#### 3.5.2. Optimization Problem of the Proposed E-LCKSVD

The first step is to generate α and β (γ=1−α−β); these two numbers are real, and the value range is 0 to 1. Then, from 15 binary numbers which are used to determine the response of the sensor which will be used to initialize the dictionary, one of the responses of the sensor will be selected, only when the corresponding binary number is 1. The classification accuracy of the E-nose is the fitness function; a set of parameters can be chosen only if its corresponding fitness function reaches the maximum. The detailed information of the parameters that needed optimization is shown in Table 4.

## 4. Results and Discussion

**Figure 5:**
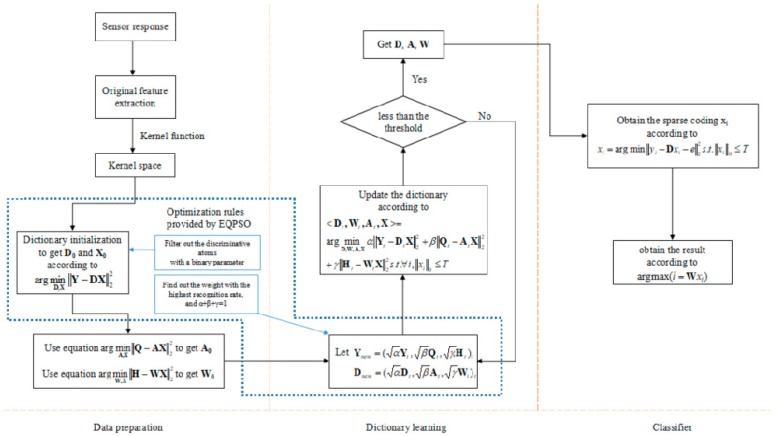
The flow chart of the proposed enhanced label-consistent KSVD (E-LCKSVD).

As mentioned before, there are 96 samples of 4 classes (24 samples in each class), and we randomly select 16 samples from each class to build the training data set (16 × 4 = 64 samples), and the rest of the samples are used to build the test data set (8 samples in each class). Figure 5 gives the work flow of E-LCKSVD. In order to verify that the kernel function can help L-KSVD deal with the data of the E-nose more effectively, we have entered several sets of comparative experiments. To determine whether to add a kernel function to the map data to a variable controlled by a high-dimensional space, we compare the processed and unprocessed data into KSVD and L-KSVD respectively. Since KSVD does not have the ability of pattern recognition by itself, we input the sparse matrix obtained by it into the extreme learning machine (ELM) [36] for pattern recognition. Each set of procedures is repeated 10 times, and the one with the highest recognition rate was taken as the final result, which is shown in Table 5. 

**Figure 6:**
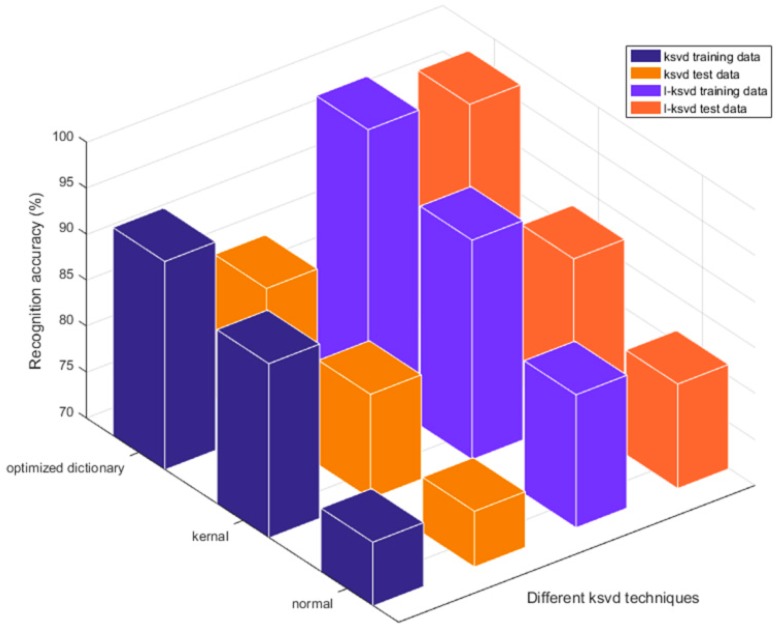
The classification accuracy of different KSVD techniques.

It can be seen obviously that the addition of the kernel function improves the discriminating precision significantly. As shown in Figure 6, KSVD combined with ELM is more effective in processing data, as does L-KSVD.

Next, we compare the dictionary initialization method proposed in this paper with the previous dictionary initialization methods. We found that the initialization of the dictionary can affect the classification accuracy. 

**Figure 7:**
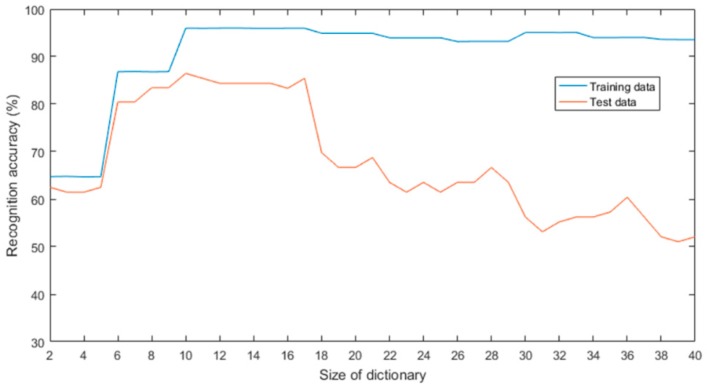
The recognition accuracy of different dictionary sizes.

As shown in Figure 7, a too small dictionary leads to the loss of information, resulting in a low recognition rate. Meanwhile, the process of training the dictionary will be complex when the dictionary is too large, and a large dictionary does not mean a high classification accuracy because the data of the sensor array is redundant. In addition, we found that even though the dimensions were the same, the result is different when different sensors are selected to initialize the dictionary, just like shown in Table 6. The classification accuracy of different dictionary initialization methods are shown in Table 7. Finally, the size of the dictionary is 14. Compared with the random initialization method, it is significant to select the initialization dictionary purposefully. The training set recognition rate of E-LCKSVD using the optimized dictionary initialization method is as high as 98.4%, and the test set recognition rate reaches 96.9%.

**Figure 8:**
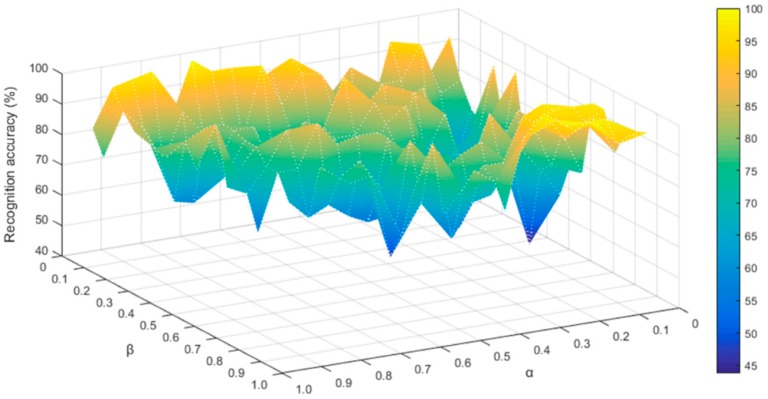
The classification accuracy of different values of α and β.

Figure 8 is the comparison of the recognition rate using several different weighted coefficients and the corresponding weight when the highest recognition rate is reached. It can be seen visually that the weights have a great influence on the effect of the whole system. Therefore, finding the appropriate weight value is an important step. Different weights combinations have the problem of the local optimal solution; we use EQPSO to avoid this, and then, we find the global optimization point: α=0.7661,β=0.0351.

Then, we compare the proposed method with the results of the existing common feature extraction and classifier in an E-nose. We use the maximum steady-state response of the original response curve, PCA (as the representative of the linear feature extraction algorithm) and KPCA (as the representative of the nonlinear feature extraction algorithm) to construct the feature matrix, and select SVM, RBFNN and K-LDA (the kernel LDA) as classifiers. The data processing results are shown in Table 8. 

From Table 8, we can find that the best result is obtained when KPCA is used to extract the feature and SVM is the classifier, but this result (96.6/90.6%) is still a little worse than E-LCKSVD (98.4/96.9%). The advantages and drawbacks of all techniques used in this paper is shown in Table 9.

## 5. Conclusions

In this paper, an E-nose is used to classify the orange juice of different storage days. An intelligent algorithm is important for the E-nose. Feature extraction and classification are two key steps, and the existing techniques generally study the two links separately, but the classifier can only adjust itself and cannot train the feature extraction in the process of training, so the result is not satisfactory. In this paper, an enhanced L-KSVD technique, E-LCKSVD, is proposed. This technique can combine feature extraction with pattern recognition and adjust and optimize the feature extraction and classification according to the training results. Aiming at the traditional L-KSVD algorithm, combined with the characteristics of the data in this paper, we have made some improvements to the algorithm: Firstly, the nonlinear response of the E-nose is mapped to linear data by using the kernel function. Then, a dictionary initialization method with the help of EQPSO is proposed to initialize the dictionary of KSVD/L-KSVD, and a KSVD/L-KSVD dictionary initialization method suitable for the characteristics of E-nose data is obtained. Finally, the weighted coefficients of different parts of the objective function of L-KSVD are standardized, the influence of weighted coefficients on the recognition rate is studied, and an optimized setting method of weighted coefficients based on EQPSO is proposed. The experiments of using E-LCKSVD to get higher recognition accuracy results are very satisfactory. In short, we conclude from a series of results that E-LCKSVD is an ideal solution for distinguishing gas data from an E-nose.

## Figures and Tables

**Table 1:** The analysis of orange juice aroma components.

No.	Compound	No.	Compound
1	Ethanal (C_2_H_4_O)	26	α-copaene (C_15_H_24_)
2	Ethyl acetate (C_4_H_8_O_2_)	27	Decanal (C_10_H_20_O)
3	Methyl butanoate (C_5_H_10_O_2_)	28	Linalool (C_10_H_18_O)
4	α-pinene (C_10_H_16_)	29	Germacrene D (C_15_H_24_)
5	α-thujene (C_10_H_16_)	30	Caryophyllene (C_15_H_24_)
6	Ethyl butanoate (C_6_H_12_O_2_)	31	P-menth-1-en-4-ol (C_10_H_18_O)
7	Butanoic acid,2-methyl-,ethyl ester (C_7_H_14_O_2_)	32	Citronellol acetate (C_12_H_22_O_2_)
8	Hexanal (C_6_H_12_O)	33	β-farnesene (C_15_H_24_)
9	β-pinene (C_10_H_16_)	34	p-menth-1-en-8-ol (C_12_H_20_O_2_)
10	β-thujene (C_10_H_16_)	35	Valencene (C_15_H_24_)
11	3-carene (C_10_H_16_)	36	Nerol acetate (C_12_H_20_O_2_)
12	α-phellandrene (C_10_H_16_)	37	(S)-carvone (C_10_H_14_O)
13	β-myrcene (C_10_H_16_)	38	1,6-octadiene,3-(1-ethoxyethoxy)-3, 7-dimethyl (C_14_H_26_O_2_)
14	α-terpinene (C_10_H_16_)	39	Cadinene (C_15_H_24_)
15	Limonene (C_10_H_16_)	40	Lavandulol acetate (C_12_H_20_O_2_)
16	β-phellandrene (C_10_H_16_)	41	1-cyclohexene-1-methanol,4-(1-methylethenyl)-,acetate (C_12_H_18_O_2_)
17	Ethyl caproate (C_8_H_16_O_2_)	42	1-octanol (C_8_H_18_O)
18	γ-terpinene (C_10_H_16_)	43	Cyclopentanecarboxylic acid,2-ethylcyclohexyl ester(C_14_H_24_O_2_)
19	β-ocimene (C_10_H_16_)	44	2,2,3,5,6-pentamethyl-3-heptene (C_12_H_24_)
20	β-cymene (C_10_H_14_)	45	α-caryophyllene (C_15_H_24_)
21	Terpinolene (C_10_H_16_)	46	1-undecanol (C_11_H_24_O)
22	Cyclopentanone,2-methyl (C_6_H_10_O)	47	1-propene 3-(2 cyclopentenyl)-2-methyl-1 (C_21_H_22_)
23	Octanal (C_8_H_16_O)	48	Dimethyl phthalate (C_10_H_10_O_4_)
24	Nonanal (C_9_H_18_O)	49	2,4-diphenyl-4-methyl-1-pentene (C_18_H_20_)
25	Octyl acetate(C_10_H_20_O_2_)	50	2,4-diphenyl-4-methyl-2-pentene (C_18_H_20_)

**Table 2:** The main sensitive characteristics of gas sensors.

Sensors	Sensitive Characteristics
TGS813	Methane, Propane, Ethanol, Isobutane, Hydrogen, Carbon monoxide
TGS816	Combustible gases, Methane, Propane, Butane, Carbon monoxide, Hydrogen, Ethanol, Isobutane
TGS822	Organic solvent vapors, Methane, Carbon monoxide, Isobutane, n-Hexane, Benzene, Ethanol, Acetone
TGS2600	Gaseous air contaminants, Methane, Carbon monoxide, Isobutane, Ethanol, Hydrogen
TGS2602	VOCs, Odorous gases, Ammonia, Hydrogen sulfide, Toluene, Ethanol
TGS2610C	Ethanol, Methane, Propane, Combustible gases, Isobutane
TGS2611E	Methane, Propane, Isobutane
TGS2620	Vapors of organic solvents, Combustible gases, Methane, Carbon monoxide, Isobutane, Hydrogen, Ethanol
MQ135A	Hydrogen, Smoke, Carbon monoxide, Ethanol
MQ135	Ammonia, Benzene series material, Acetone, Carbon monoxide, Ethanol, Smoke
MQ136	Hydrogen sulfide, Sulfur dioxide
MQ137	Ammonia, Trimethylamine, Ethanolamine
MS1100	Formaldehyde, Benzene, Toluene, Xylene, Aromatic compound
MP4	Methane, Combustible gases, Biogas, Natural gas
MP503	Smoke, Isobutane, Formaldehyde, Ethanol

Note: the response of these sensors is nonspecific. Besides their main sensitive gas in Table 2, they are also sensitive to other gases.

**Table 3:** The classification accuracy of different weighted coefficients.

No.	α	β	γ	Acc_Train (%)	Acc_Test (%)
1	0.6	0.3	0.1	92.2	87.5
2	0.3	0.6	0.1	89.1	84.4
3	0.1	0.3	0.6	84.4	75.0
4	0.33	0.33	0.33	90.6	84.4

**Table 4:** Details of the parameters needing optimization.

1. Weighted Coefficient	2. Binary Number for Dictionary Initialization
α,β and γ α + β + γ = 1, and α, β, γ∈(0,1).	$b_{1},b_{2},\cdots ,b_{15}$, The value of $b_{n},n=1,2,\cdots ,15$ is 0 or 1.

**Table 5:** The classification accuracy of different KSVD techniques (%).

	Original Data		Kernel Data	
	KSVD+ELM	L-KSVD	K-KSVD+ELM	E-LCKSVD
**Acc_train**	76.9	84.4	88.9	93.8
**Acc_test**	76.0	81.3	81.3	87.5

**Table 6:** The classification accuracy when different sensor responses are chosen to initialize the dictionary.

	Sensors	Binary Number								
1	TGS813	1	0	0	1	1	1	1	1	0
2	TGS816	1	1	0	1	1	1	1	0	1
3	TGS822	1	0	1	1	1	0	1	1	0
4	TGS2600	1	1	1	0	0	1	0	1	1
5	TGS2602	1	0	1	1	1	1	0	1	1
6	TGS2610C	1	1	1	1	0	0	1	1	1
7	TGS2611E	1	0	1	0	1	1	0	1	1
8	TGS2620	0	1	0	1	0	1	0	0	1
9	MQ135A	1	0	0	0	1	1	1	1	1
10	MQ135	1	1	0	0	1	1	1	1	1
11	MQ136	0	1	1	0	1	1	1	1	0
12	MQ137	0	1	1	1	0	1	1	0	0
13	MS1100	0	1	1	1	1	0	0	0	0
14	MP4	1	1	1	1	0	0	1	0	1
15	MP503	0	1	1	1	1	0	1	1	1
**Acc_train (%)**		90.6	92.2	89.0	98.4	90.6	81.3	93.8	98.4	85.9
**Acc_test (%)**		81.3	84.4	87.5	93.8	71.9	75.0	87.5	96.9	81.3

**Table 7:** The classification accuracy of different dictionary initialization methods (%).

	Normal Dictionary Initialization		Optimized Dictionary Initialization	
	K-KSVD+ELM	E-LCKSVD	K-KSVD+ELM	E-LCKSVD
**Acc_train**	88.9	93.8	92.6	98.4
**Acc_test**	81.3	87.5	85.4	96.9

**Table 8:** The results of the existing common feature extraction techniques and classifiers (%).

		No-Dealing	PCA	KPCA
SVM	Acc_train	92.8	94.8	96.6
	Acc_test	81.3	93.8	90.6
RBFNN	Acc_train	89.5	91.0	94.8
	Acc_test	86.5	88.5	89.6
K-LDA	Acc_train	93.3	93.7	96.0
	Acc_test	88.5	86.5	88.5

Note: No-dealing means the feature matrix containing the maximum value of the steady-state response of sensors is put into the classifiers (SVM, RBFNN and K-LDA) directly.

**Table 9:** The advantages and drawbacks of all the techniques used in this paper.

	Advantages and Drawbacks
PCA	This linear feature extraction algorithm obtains the new feature according to the variance contribution rate, but the effect is not satisfactory when dealing with the nonlinear data.
KPCA	With the help of the kernel function, the data can be mapped to a high-dimension space and then analyzed by PCA, which has the ability of processing the nonlinear data, but the high-dimension mapping increases the computational complexity.
K-LDA	LDA is a kind of supervised linear classifier. With the help of the kernel function, it has the ability to classify the nonlinear data to some extent, but the improvement of the recognition rate of the kernel function is limited.
RBFNN	An artificial neural network used in an E-nose earlier: using a radial basis function as the nonlinear mapping function, the recognition rate is better than K-LDA, but still lower than SVM.
SVM	For a long time, SVM is considered as an optimal classifier. With the help of the kernel function, SVM has an excellent ability to process data, but the recognition rate is affected by the quality of the input data.
E-LCKSVD	The feature extraction and classifier are integrated into one, considering the influence of the dictionary initialization, kernel function and weight coefficient of the objective function on the recognition rate; if the idea of semi-supervised learning can be added, it would be more valuable to use unlabeled data which is cheap and easily available.
